# Let us integrate sexual health—do psychiatrists integrate sexual health in patient management?

**DOI:** 10.1007/s00737-019-01016-9

**Published:** 2020-01-02

**Authors:** Tamara Seitz, Lucia Ucsnik, Andrea Kottmel, Johannes Bitzer, Bela Teleky, Henriette Löffler-Stastka

**Affiliations:** 1 Department of Infectious Diseases and Tropical Medicine, SMZ SÜD, Vienna, Austria; 2 Department of Visceral Surgery, University Clinic of Surgery, Medical University Vienna, Vienna, Austria; 3 Private Practice for Gynecology and Sexual Medicine, Vienna, Austria; 4 Private Practice for Gynecology, Basel, Switzerland; 5 University Clinic of Psychoanalysis and Psychotherapy, Medical University Vienna, Währinger Gürtel 18-20, 1090 Vienna, Austria; 6 Austrian Society of Psychiatry, Psychotherapy and Psychosomatics, Section Psychotherapy, Vienna, Austria; 7 Postgraduate Unit, Teaching Center, Medical University Vienna, Vienna, Austria

**Keywords:** Sexual history, Sexual health, Sexual dysfunction, Sexual problems, Sexual medicine

## Abstract

The high prevalence of sexual dysfunction and the importance of sexual health issues in general stress the need for a physician to integrate sexual health issues in patient management. In this study, we evaluate the frequency of psychiatrists addressing sexual health issues as well as their attitude towards sexual health. Plus, we want to evaluate the multi-professional network for patient treatment that is needed by physicians for collaboration with other medical specialists and health care professionals. At total 100 psychiatrists (age range 30–60 years), participating at the annual meeting of the Austrian Society of Psychiatry, Psychotherapy, and Psychosomatics, were invited to self-assess their caring for patients’ sexual health issues and answer a self-report questionnaire. The return rate was 74%. A third of the participating psychiatrists and psychotherapists stated to address sexual health in patients in daily routine. Twenty-five percent of the physicians suspected sexual health problems in 60–100% of their patients but did not ask the patients about this topic. Mentioned reasons why patients would not actively address sexual problems were rated by the survey participants “a different problem was more important”, “lack of time”, and “embarrassment”. Only few of the participating psychiatrists stated to offer a consultation in sexual health to the patients, none to offer sexual therapy. A mentioned reason was “lack of competence regarding sexual health”. Twelve percent referred the patients with sexual issues to a physician with another medical specialization, especially to experts in gynaecology and obstetrics, to experts in urology, or to andrologists. However, a need for a network in the field of sexual medicine was stated and an unawareness of a sexual health care network: where to refer the patients in need. Our data showed an increased need in the routine treatment and management of sexual health care in psychiatrists and psychotherapists. Plus, the data stresses the need for professional sexual medicine qualification and for extended cooperation between different medical fields and health care professionals in order to integrate sexual health topics professionally in daily routine.

## Introduction

Sexual health is essential for general health, quality of life, and well-being. The Declaration for Sexual Rights by the World Association for Sexual Health (WAS 2014) states that sexual health is a right grounded in universal human rights. Sexual health is a state of physical, emotional, mental, and social well-being in relationship to sexuality. It is not merely the absence of disease, dysfunction, or infirmity. Sexual health requires a positive and respectful approach to sexuality and sexual relationships, as well as the possibility of having delightful and safe sexual experiences, free of coercion, discrimination, and violence (WAS 2014). In a large survey (Mitchell et al. 2016) 38.2% of sexually active men and 22.8% women reported about sexual problems. Thus, there is a need for health care professionals to integrate sexual health issues in patient management and treatment (Nusbaum and Hamilton 2002).

**Fig. 1:**
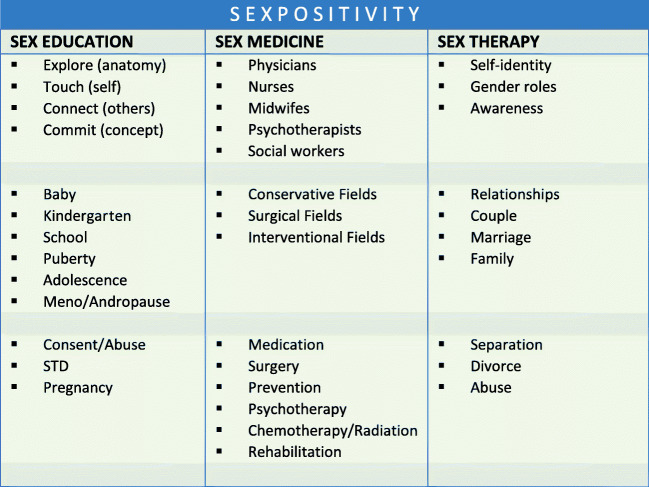
The three columns of sex-positivity

Sexual health and sex-positivity is supported by three different fields, contributing to a holistic sex-positivity (see Fig. 1):

- Sexual pedagogic: taking care of sexual health issues in the course of the life-span and supporting the process of coping with it
- Sexual medicine: taking care of diseases as well as medical diagnostic and therapeutic procedures (conservative, surgical, interventional) causing sexual dysfunctions or change of sexual health as a symptom and as a predictor for diseases
- Sexual therapy: consulting patients in times of troubles due to personal as well as sexual identity, gender roles, concepts of relationships, living together as well as separating, and its impacts on sexual health

Nowadays, health care professionals tend to mix these three fields up into one, named “sexology”. They refer patients to psychiatrists and psychotherapists in order to take care of the topic of sexual health, as if the topic is only a “psychic issue” and the need for a “talk”. Anyway, medical aspects including sexual health issues should be taken seriously and covered by various medical specialists and experts working together in a sexual health care network consisting of different disciplines.

In order to provide and meet patients’ needs in terms of sexual health, it is important that every health care professional and physician estimates the impact on sexual health, whenever taking care of the diseases treated and therapy prescribed. Management and integration of the topic of sexual health in daily routine is necessary.

Furthermore, it is important to build a multi-disciplinary and multi-professional network for sexual health care in order to refer patients to the expert needed.

As it also has been shown that sexual satisfaction correlates with a higher quality of life (Flynn et al. 2016) and longevity (Palmore 1982), a doctor’s aims may be

- to explore and manage the patients’ sexual health and address pre-existing sexual problems,
- to inform (e.g. about possible reasons for sexual dysfunction like drugs’ side effects causing or worsening sexual dysfunction), and
- to prevent (e.g. sexual dysfunction, STD, or unwanted pregnancy).

Due to the importance of this topic and the lack of literature, the Igls-Vienna-SexMed-Survey-Program was started at the Medical University of Vienna. It evaluates via a 24-item self-assessment-questionnaire whether various health care professionals and specialist in Austria (but also in whole Europe) do address the topic of sexual health. It examines how the network and landscape of patient treatment in terms of sexual health are drawn and which services are available for patients. Furthermore, it evaluates the professional qualification in the field of sexual health and sexual medicine. The study started in the field of coloproctology; in this study, we focus on psychiatrists.

Reconsidering the lack in the literature, our objective for this paper is to evaluate

1. the self-assessed frequency of psychiatrists addressing sexual health aspects and sexual dysfunction
2. their attitude towards sexual health
3. the existing professional network of referral in terms of sexual health

The further aim of this study is to evaluate the existing landscape of patient-care in terms of sexual health as well as the need for change and adaption.

This study is an important part to build a multi-disciplinary and multi-professional network for sexual health. This network should provide professional state-of-the-art patient management and care in case of sexual health issues, problems, dysfunction, or need for sex-education, sexual medicine, and sexual therapy.

## Material and methods

For investigation, the authors chose a hotspot recruitment and a hypothesis-generating design in order to display naturalistic data to close the practice-oriented research gap. The survey was conducted in April 2017 within the annual meeting of the Austrian Society for Psychiatry, Psychotherapy, Psychosomatics (ÖGPP), Section Psychotherapy, which took place in Austria (Gmunden, OÖ). The aim was to assess Austrian and Middle European psychiatrists’ own perception concerning their current routine care for patients with sexual dysfunctions and their attitudes towards patients’ sexual health issues in daily routine. On purpose, we did not perform an analysis based on epidemiological data due to recruitment of participants and data available at that point of survey.

The survey was conducted with a self-administered questionnaire (SAQ) including 24 items with open answers, multiple-choice answers, and visual analogue scales. The questionnaire, developed and published by Kottmel et al. (2014), is 3-parted:

- Part A included questions about the physicians’ patients and their behaviour and needs regarding sexual health issues.
- Part B was about the physician’s therapy offers regarding sexual health.
- Part C dealt with the physician’s profile.

Seventy-four percent of the 100 psychiatrists participated and returned the questionnaire within the annual meeting. The study was approved by the ethics committee of the Medical University Vienna and all participants gave their informed consent to take part in the study (EK Nr. 1360/2017).

### Characteristics of interviewed psychiatrists

Out of 100 questionnaires, 74 were returned. Of the respondents, 32.4% (*n* = 24) were male and 67.6% (*n* = 50) female. The age of the participants ranged between 30 and 60 years, whereby the majority (*n* = 25) were between 40 and 50 years old. Sixty-three percent (*n* = 46) have already been working as psychiatrists for 10–20 years, 16.4% (*n* = 12) between 5 and 10 years, 11% (*n* = 8) between 2 and 5 years, and 9.6% (*n* = 7) over 20 years. One percent did not answer this question. The majority, 43.8% (*n* = 28), were also psychotherapists since 10–20 years, 28.1% (*n* = 18) for 5–10 years, 17.2% (*n* = 11) between 2 and 5 years, and 9.4% (*n* = 6) less than 2 years, 1.6% (*n* = 1) more than 20 years. This shows that the majority of participants were highly experienced in the field of psychiatry as well as psychotherapy. Only physicians with completed training for the specialisation in psychiatry were included, e.g. experts in internal medicine were excluded. 97.1% (*n* = 66) were Austrian; 6 participants did not answer the question.

### Statistics

Descriptive statistics were calculated and effects of gender, age, years of working experience as psychiatrist, and attendance of sexual health training courses were controlled. Ordinal variables were compared between two groups with Wilcoxon rank sum test, between 3 or more groups with Kruskal Wallis test. To estimate the correlation between two ordinal variables Kendall’s tau was calculated and to document the relationship between categorical variables *χ*^2^ test was used.

No correlation for multiple testing was applied: significance level was set to *α* = 0.05 for all tests. Therefore, all presented *p* values have to be interpreted with caution and hypotheses generating only. No statistical test was calculated in case of small sample sizes (less than 4 per group in a 2-group comparison, less than 10 observations for ordinal variables). The analysis was carried out with *R* due to advice of the Institute for Medical Statistics of the Medical University Vienna.

## Results

### Part A—Frequency of addressing sexual health

**Fig. 2:**
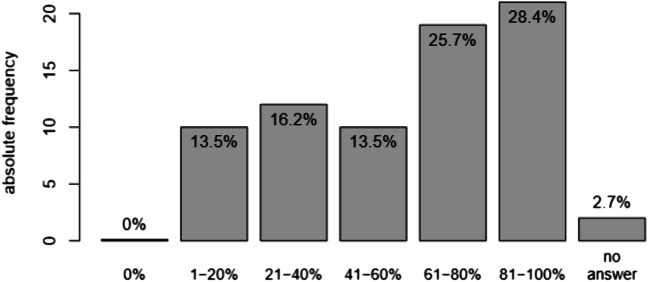
Percentage of patients routinely asked about sexual health by surveyed psychiatrists

The percentage of patients which are routinely asked about sexual health issues as stated by the participating psychiatrists is shown in Fig. 2. No influence of gender of the psychiatrist was found. However, physicians with training in sexual health issues showed a significantly higher rate of asking their patients about sexual health. The rate of asking about sexual health was shown to increase with years of experience in the specialty (*p* < .001) and also age of psychiatrists (*p* < .001).

**Fig. 3:**
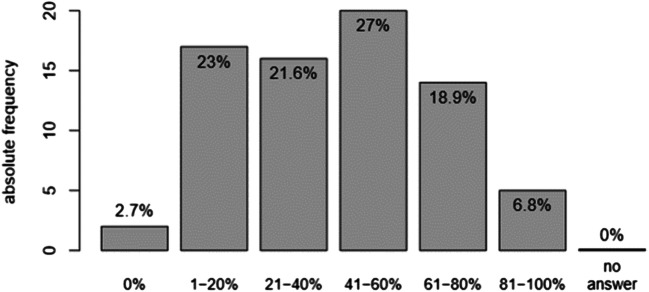
Percentage of patients, where sexual health issues are suspected but were not addressed by the attending psychiatrists

Figure 3 shows the percentage of patients, in whom the attending psychiatrists suspected sexual health problems, but did not address and ask the patients. There was no influence in age or gender of the participating psychiatrists. The more experience the psychiatrists had in the field and discipline of psychiatry (*p* < 0.05), the more the experts were specialised in sexual health issues (*p* < 0.05), the more likely the psychiatrists did address sexual problems when suspecting them.

**Fig. 4:**
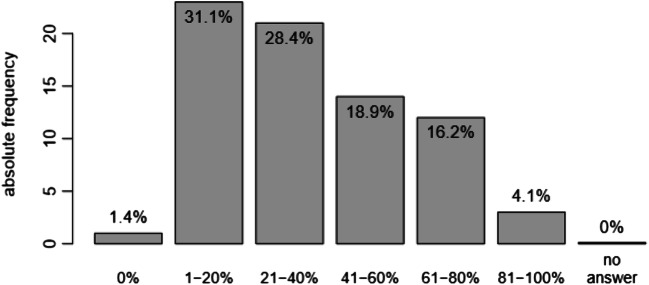
Percentage of patients bringing up the topic sexual problem on their own while talking to a psychiatrist

Figure 4 shows the percentage of patients bringing up the topic of sexual problems on their own while talking to a psychiatrist. Neither gender nor age of the psychiatrists showed to have influence on the frequency of patients bringing up the topic by their own. However, there was a significant relationship between the frequency of patients raising the topic and years of experience of the psychiatrists in psychiatry (*p* < 0.001), as well as specialisation in sexual medicine (*p* < 0.05).

### Reasons for not addressing sexual health issues in psychiatrists’ daily routine

In Table 1, the reasons of psychiatrists are listed to give a picture of why they assume that patients do not address sexual health problems more often. The leading statement was “a different problem was more important”, followed by “lack of time” and “embarrassment [of the patients] to talk about this topic”.

**Table 1:** Stated reasons by psychiatrists not to address sexual health issues

	*N*	%
Lack of time	27	38.6
Other problems more important	40	57.1
Embarrassing topic	12	17.1
Language barrier	8	11.4
Age	8	11.4
Religion	8	11.4
Culture	7	10
Other, to be specified	0	0

### Part B—Management of sexual health care

Table 2 shows the recommendations regarding consultation or/and therapy for sexual health issues. The most often statement mentioned (45.7%) was “evaluation of pre-existing medication which could cause sexual dysfunction”. None stated to offer sexual therapy. Nine (12.9%) were referring patients with problems regarding sexual health to a physician with another medical specialization, not specified.

**Table 2:** Therapy offers regarding sexual health issues

	*N*	%
Evaluation of drugs having an impact on sexual dysfunction	32	45.7
Psychotherapy	28	40
Pain therapy	18	25.7
Couple talk	11	15.7
Couple therapy	11	15.7
Referral to specialists	9	12.9
Hormone therapy	4	2.9
Information on physiological sexual function	1	1.4
Sexual medicine	1	1.4
Evaluation of anticoagulation therapy	1	1.4
Sexual therapy	0	0

In case of referring patients to physician with another medical specialization, 52.7% (*n* = 39) of the psychiatrists stated to refer to gynaecologists and obstetricians, 32.4% (*n* = 24) of the participants to experts in internal medicine, 52.7% (*n* = 39) to urologists, 32.4% (*n* = 24) to andrologists and 31.1% (*n* = 23) to psychotherapists proper. Only 5.4% (*n* = 4) referred the patients to a physician specialized in sexual medicine.

### Aspects reducing success of therapy

Fifty-nine percent (*n* = 41) of the psychiatrists stated that in general, their patients “often” took and followed their advice. Forty-two of the participating psychiatrists (59.3%) reported in the self-assessment they could help 21–40% of the patients themselves, 26.9% (*n* = 12) each 1–20% and 41–60% of the patients, 4.2% (*n* = 3) 61–80% of the patients and 2.8% (*n* = 2) 81–100% of the patients.

Aspects mentioned by psychiatrists that reduce success of treatment are listed in Table 3. The most often stated aspects (33.8%) were “lack of patients’ motivation for therapy” and “lack of improvement after therapy/intervention”.

**Table 3:** Reasons, in the opinion of asked psychiatrists, for treatment failure

	*N*	%
Patients’ lack of motivation	24	33.8
No specialist known for referral	23	32.4
Lack of improvement/recovery after treatment	20	28.2
Patients’ culture	15	21.1
Patients’ sexual orientation	12	16.7
Lack of professional, sexual medicine competence	9	12.7
Patients’ age	9	12.7
Patients’ religion	9	12.7
Lack of time	8	11.3
Patients’ nationality	3	4.2
Own age	1	1.4
Own religion	0	0
Own culture	0	0
Own nationality	0	0
Own sexual orientation	0	0

## Discussion and conclusions

Our study showed that less than a third of the psychiatrists participating in this survey reported in the self-assessment that they ask over 80% of their patients about their sexual health issues.

About the half of the participants suspected sexual problems in 41–80% of the patients and did not bring up the topic. About 7% of the psychiatrists stated to assume sexual health problems in over 80% of the patients and did not talk about the topic with their patient.

The mentioned main reason (38.6%) for not addressing sexual health by patients was the impression of the psychiatrists that other problems were more important for the patients. Anyway, literature showed that physicians tend to have a lack of understanding about the importance of sexual health issues (Morand et al. 2009; Nusbaum and Hamilton 2002) and underestimate the prevalence severely (Abdolrasulnia et al. 2010; Nusbaum and Hamilton 2002; Papaharitou et al. 2008).

About 38.6% stated that “lack of time” was another main reason why not addressing sexual health issues. Furthermore, our data showed a lack of offers and abilities of recommendation for consultation and treatment of sexual problems even if sexual issues are addressed. Less than half of psychiatrist evaluated the patient’s medication regarding side effects causing sexual dysfunction: a third stated that other medication such as antidepressants has impact on patients’ sexual health. Forty-five percent estimated that prescribed medication could cause sexual dysfunction. Only 12.9% gave information about the physiology of sexual function. None was offering sexual therapy.

Anyway, only 12.9% of the participating psychiatrists were referring the patients with sexual health issues to a physician with another medical specialization. Only 5.4% referred the patients to a physician specialized in sexual medicine.

The majority (52.7%) of the psychiatrists referred the patients to an expert in gynaecology and obstetrics assuming that this profession takes care of sexual health issues. However, Kottmel et al. (2014) showed that in Switzerland, only 8% of the gynaecologists were addressing the topic of sexual health regularly. One of the reasons mentioned in this study—of not to address the topic more often—was time constraints. Although we did not find data about Austrian gynaecologists, a similar situation in Austria can be assumed. This shows the contradicting expectations and prejudices of health care professionals which medical speciality is supposed to be qualified and to take care of sexual health care.

This stresses the importance to adapt health care professionals’ expectations and prejudices. Plus, an extention of the network of medical professionals dealing with sexual health issues is crucial. This network should deal with the three important pillars of sexual health: Sexual Education, Sexual Medicine, and Sexual Therapy. Sexual Education should cover the whole life-cycle: children at kindergarten and school to adults, the changes in puberty, adolescence up to andro- and menopause as well as seniors dealing with changes in sexual health.

On one hand, a positive attitude towards sexuality, so called sex-positivity, should be emphasised, on the other hand, information about consenting sexual activity, preventable STDs, unwanted pregnancy should be given but not as the only information about sexual education.

Sexual Medicine and Therapy do not only affect medical specialities (e.g. sexual dysfunction after operation or as side effect from medication or therapy and interventions) but also paramedical professions, such as nurses (pain, continence and ostomy, oncology), mid-wives, and clinical psychologists. It is from utmost importance to create a well working network of patient management in the field of sexual health among different professional groups and medical specialities, including teachers and police. Often, it is automatically believed that psychiatrists do offer consultation about sexual health issues at all. This survey showed that this prejudice is false.

Therefore, it is necessary to create adequate information about the offer of sexual health care for physicians, professional groups dealing with sexual health and patients. All professional groups should always know to whom patients to refer to or ask for information in case the challenges faced exceed one’s professional competence. Furthermore, patients should easily get information where to get help in case of troubled sexual health.

Another interesting finding in our study is that the physician’s embarrassment is an often-mentioned reason for not addressing sexual health. It is a well-known phenomenon in literature (Morand et al. 2009; Nusbaum and Hamilton 2002). It has been shown that physicians who are comfortable talking about sexual health reveal and contain sexual health problems more likely (Bachmann et al. 1989; Burnap and Golder 1967). A possible reason often discussed in literature is the physician’s feeling of being inadequately trained (Bitzer et al. 2013a; Coverdale et al. 2011; Nusbaum and Hamilton 2002; Parish and Clayton 2007; Tsimtsiou et al. 2006; Waineo et al. 2010). In our study, 12.7% (*n* = 9) considered the lack of sexual health competence as a reason for reduced success of treatment. Other reasons mentioned were age, religion, and culture of the patient. In several other studies, physicians reported gender or culture differences between doctor and patient as a source of difficulty in Sexual History Taking (Burd et al. 2006; Löffler-Stastka et al. 2016; Temple-Smith et al. 1996) which was confirmed by this survey, too.

Another finding in our data that stresses the importance of adequate training is that physicians with increased experience and training in sexual medicine tend to address sexual health problems more often and patients feel more comfortable bringing up the topic by their own.

Therefore, the idea is emphasized that training for sexual health care should start during medical school at medical university. For example, case-based e-learning programs and simulated patient (SP) contact might be implemented. Current literature demonstrates that case-based e-learning and especially training with SPs in medical education are valuable tools (Levine and Swartz 2008; McNaughton et al. 2008), correlating with a high learners’ satisfaction (Himmelbauer et al. 2018; Seitz and Löffler-Stastka 2015) and an improvement in learners’ understanding of certain topics and skills (Xie et al. 2015). The SP might play different roles in various settings, so the student learns sexual health care in different medical fields like internal medicine, obstetrics, or psychiatry. In these training, difficult topics like gender difference, foreign religion, or barrier of language communication could be discussed. The next step would be to increase the awareness and positive attitude among physicians regarding sexual health issues and offer a wide range of information and training possibilities. Several publications are dealing with training possibilities for physicians to manage about sexual health (Alder et al. 2007; Bitzer et al. 2013b; Laan et al. 2013; Seitz et al. 2016; Tsimtsiou et al. 2006; Wittenberg and Gerber 2009).

Twenty-three doctors (31.2%) stated they were asked by only 1–20% of the patients, 47.3% (*n* = 21) by 21–60% and 20.3% by 61–80% of the patients about sexual health issues. This finding is not surprising: it has been reported that patients prefer that their doctors initiate the subject (Meystre-Agustoni et al. 2011).

Our data show there was no gender-related difference regarding frequency of addressing sexual problems. This is in contrast to other publications, showing that female physicians more likely and more frequently take sexual history than men (Löffler-Stastka et al. 2016; Wimberly and Hogben 2004). An explanation might be that a significantly higher percentage of participants in our study were females leading to an inaccuracy in calculation.

The study also has some limitations:

The main two are on one hand the self-assessment of percentages of patients asked by doctors in part A of the questionnaire which does not picture epidemiologic data but the participants’ awareness and guessing. On the other hand, there is a selection bias resulting from the use of a SAQ. Another limitation is the unequal gender distribution. A third limitation is that not all members of the Austrian Society of Psychiatry, Psychotherapy and Psychosomatics were asked. Only the members who participated at the annual congress were addressed. They are probably more open to life-long learning and changing their practice. Furthermore, our sample included especially psychiatrists with a long working experience and out-patient practice. However, the study’s strength is the high response rate of 74% reducing the biases rate considering that the non-responders might be less interested in the issue of sexual health.

Over 70% were working in this field for more than 10 years. Thus, the sample might be not representative for all Austrian psychiatrists.

Further studies are necessary to get a complete insight of the existing landscape of patient-care in terms of sexual health. Different health care professionals and physicians from different medical specialization should be asked. Plus an evaluation about patient’s wishes and needs regarding consultation and therapy of sexual health issues is necessary.

To sum up, we could show that though most psychiatrists think that the vast majority of their patients struggle with sexual problems, they do not address sexual health issues in daily routine patient treatment and patient-consultation. Interestingly, none of the participating psychiatrists and psychotherapists offered sexual therapy, contrary to expectations of surveys done with other medical specializations and health care professionals and soon to be published.

An improvement might be obtained with an extension of sexual health care network between different medical specializations and health care professionals as well as various training possibilities for physicians.

Furthermore, the general awareness of the importance of sexual health must be raised and broader information should be provided about the local offers regarding sexual medicine.
